# Magnolol and honokiol: potential lead compounds for the new drug discovery in treating autoimmune diseases

**DOI:** 10.3389/fphar.2025.1578971

**Published:** 2025-04-23

**Authors:** Xian Lin, Jian Chen

**Affiliations:** ^1^ Department of Rheumatism and Immunology, Peking University Shenzhen Hospital, Shenzhen, China; ^2^ Institute of Immunology and Inflammatory Diseases, Shenzhen Peking University-The Hong Kong University of Science and Technology Medical Center, Shenzhen, China; ^3^ Shenzhen Key Laboratory of Inflammatory and Immunology Diseases, Shenzhen, China

**Keywords:** magnolol, honokiol, autoimmune diseases, rheumatoid arthritis, lead compound

## Introduction

Autoimmune diseases (AIDs) are a series of diseases caused by the reduced or destroyed immune tolerance of the immune system to its own components due to some reasons, resulting in auto-antibodies or (and) sensitized lymphocytes damaging its own organs and tissues (Balogh et al., 2024). Clinically, AIDs are categorized into organ-specific and systemic types. Organ-specific AIDs include inflammatory bowel disease (IBD), lupus nephritis (LN), multiple sclerosis (MS), ankylosing spondylitis (AS), IgA nephropathy (IgAN), type 1 diabetes (T1D), autoimmune liver disease (AILD), and autoimmune thyroiditis (AIT). Although termed “organ-specific”, these disorders often involve damage to multiple tissues and organs. Systemic AIDs, on the other hand, include rheumatoid arthritis (RA), systemic lupus erythematosus (SLE), scleroderma, Sjögren’s syndrome (SS), and so on. At present, there are many kinds of treatment methods for AIDs with obvious advantages and disadvantages. Thus, it is still urgent to explore effective methods to control AIDs.

**FIGURE 1:**
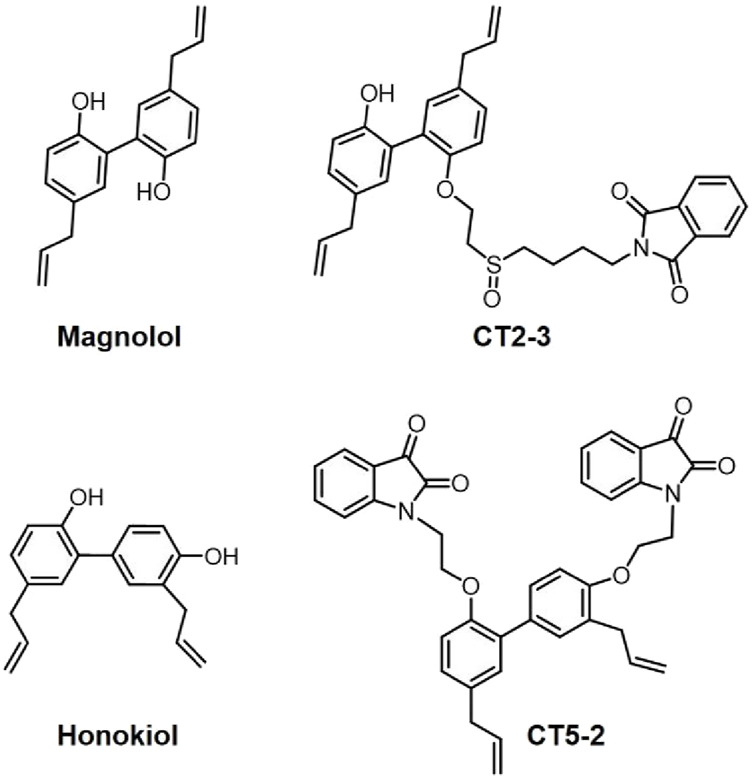
Structures of Magnolol, honokiol, CT2-3, and CT5-2.

Natural compounds, known for their structural diversity and broad biological activities, serve as a critical resource for drug discovery as well as lead compound development, playing a pivotal role in innovative drug research. Historically, scientists have made significant strides in pharmacology, drug formulation, and novel drug development. Notably, Chinese scientist Tu Youyou was awarded the 2015 Nobel Prize in Physiology or Medicine for her groundbreaking work on artemisinin (Qinghaosu), highlighting the immense potential of natural compounds in global drug development (Gao et al., 2024). Magnolol and honokiol (Figure 1), two isomeric neolignans, represent the key bioactive constituents of *Magnolia officinalis*, and have garnered increasing attention from researchers due to their wide-ranging biological properties, including anti-inflammatory, anti-cancer, anti-bacterial, anti-fungal, hepatoprotective, cardiovascular protective, neuroprotective, anti-diabetic, anti-viral, and anti-oxidant effects (Dai et al., 2024). Currently, there is no documented evidence of human toxicity associated with the herbal formulations of magnolol and honokiol (Dai et al., 2024). Moreover, these compounds are also commonly incorporated into consumer products such as mints, toothpaste, and chewing gum (Dai et al., 2024). Consequently, structural modifications of magnolol and honokiol may represent a viable strategy for developing novel therapeutics for AIDs.

## Magnolol, honokiol and their derivatives for the treatment of common AIDs

### IBD

IBD is an inflammatory bowel disease. Recent study by Zhao et al. have demonstrated that magnolol alleviates inflammation in dextran sulfate sodium (DSS)-induced colitis by restoring altered tryptophan metabolism (Zhao et al., 2017). Similarly, honokiol has been found to mitigate IBD symptoms by enhancing endothelial barrier function via its interaction with the TRPV4 channel (Niu et al., 2024). These findings suggest that magnolol and honokiol have therapeutic potential for IBD.

### LN

LN, an immune-complex-mediated kidney disease associated with systemic lupus erythematosus (SLE), significantly contributes to patient mortality and morbidity. Research has indicated that magnolol exhibits protective properties against LN progression by modulating the NLRP3 inflammasome as well as suppressing the NF-κB pathway (Huang et al., 2017). Similarly, honokiol has demonstrated therapeutic potential in LN. Studies by Ma et al. revealed that honokiol alleviates renal damage in MRL/lpr mice and disrupts pathological interactions between renal macrophages and tubular epithelial cells in LN by inhibiting the NLRP3/IL-33/ST2 signaling axis (Ma et al., 2023).

### MS

MS is a chronic inflammatory disorder marked by immune-mediated demyelination in the central nervous system. In a 2023 study, Chen et al. revealed that magnolol reduced body weight loss and disease severity in experimental autoimmune encephalomyelitis (EAE) mice, a model for MS. Their findings indicated that magnolol specifically suppresses T helper 17 (Th17) cell differentiation and cytokine production by selectively inhibiting STAT3 signaling, leading to an altered Th17/Treg cell balance (Chen J. Y. et al., 2023). This highlights magnolol’s potential as a novel STAT3 inhibitor for MS treatment.

### T1D

T1D stems from pancreatic beta cell destruction by islet reactive immune cells. In 2022, Yang demonstrated that magnolol significantly improved neurological impairments in type 1 diabetic mice (Yang et al., 2022). Similarly, honokiol has been shown to alleviate myocardial ischemia/reperfusion injury in type 1 diabetic rats by mitigating oxidative stress and apoptosis via activation of the SIRT1-Nrf2 pathway (Zhang et al., 2018). These findings highlight the therapeutic potential of magnolol and honokiol in managing T1D-related complications.

### RA

RA is a persistent autoimmune disorder that can result in disability, marked by progressive joint deterioration and symptoms beyond the joints. Studies have indicated that honokiol showed anti-inflammatory properties in RA and might act as a promising suppressor of TNF-α-triggered inflammatory factor expression in RA synovial fibroblasts (Sun et al., 2022; Li et al., 2011). Interestingly, our group successfully synthesized a magnolol derivative, namely, CT2-3 (Figure 1) that had the ability to induce cell cycle arrest and apoptosis in RA-fibroblast-like synoviocytes (FLSs) via modulating the PI3K/AKT pathway (Chen J. et al., 2023). Moreover, we also designed and synthesized a honokiol derivative (isatin-honokiol hybrid) namely, CT5-2 (Figure 1) that inhibited proliferation and triggered cell cycle arrest and apoptosis of RA-FLSs through modulation of the c-Myc/CDCA7/p65 pathway (Chen et al., 2022). These studies indicated that CT2-3 and CT5-2 may serve as promising lead compounds for the research and development of new anti-RA drugs.

## Discussion

Currently, over 100 AIDs have been identified that collectively contribute to a significant worldwide disease burden (Lenti et al., 2022). While the exact causes and mechanisms of AIDs remain unclear, they are often spontaneous or idiopathic, and most lack definitive diagnostic markers. Extensive research indicates that the onset and progression of AIDs are strongly associated with factors such as genetic predisposition, infections, hormonal imbalances, smoking, educational background, and the use of certain medications. Recent studies further classify AIDs as a significant subset of chronic inflammatory conditions. Additionally, AIDs are closely linked to the development of various cancers, making them a critical risk factor for tumorigenesis. Over the past decades, the development of new drugs to effectively control AIDs is still one of the hot spots of scientists. However, the journey of discovering new drugs is fraught with numerous obstacles and challenges. To guarantee the safety and effectiveness of medications for patients, the process involves an extensive screening phase aimed at identifying promising compounds while discarding those with significant adverse effects. Consequently, lead compounds play a pivotal role in the discovery of innovative drugs. Amidst these challenges and opportunities, natural compounds offer significant benefits owing to their diverse structures and broad biological activities. Enhanced research in this area is expected to drive the development of novel drugs with proprietary intellectual property. As previously outlined, magnolol and honokiol demonstrate diverse biological activities in treating AIDs, including IBD, MS, LN, T1D, RA, et al., positioning them as promising candidates for structural optimization. Notably, their derivatives, CT2-3 and CT5-2, have shown significant efficacy in managing RA, laying the groundwork for the development of innovative drugs for AIDs based on magnolol and honokiol frameworks.
